# Predictive Value of BRCA1, ERCC1, ATP7B, PKM2, TOPOI, TOPΟ-IIA, TOPOIIB and C-MYC Genes in Patients with Small Cell Lung Cancer (SCLC) Who Received First Line Therapy with Cisplatin and Etoposide

**DOI:** 10.1371/journal.pone.0074611

**Published:** 2013-09-13

**Authors:** Niki Karachaliou, Chara Papadaki, Eleni Lagoudaki, Maria Trypaki, Maria Sfakianaki, Anastasios Koutsopoulos, Dimitris Mavroudis, Efstathios Stathopoulos, Vassilis Georgoulias, John Souglakos

**Affiliations:** 1 Laboratory of Tumour Cell Biology, School of Medicine, University of Crete, Heraklion, Crete, Greece; 2 Department of Medical Oncology, University General Hospital of Heraklion, Crete, Greece; 3 Department of Pathology, University General Hospital of Heraklion, Crete, Greece; Mizoram University, India

## Abstract

**Background:**

The aim of the study was to evaluate the predictive value of genes involved in the action of cisplatin-etoposide in Small Cell Lung Cancer (SCLC).

**Methods:**

184 SCLC patients’ primary tumour samples were analyzed for *ERCCI, BRCA1, ATP7B, PKM2* *TOPOI, TOPOIIA*, *TOPOIIB* and *C-MYC* mRNA expression. All patients were treated with cisplatin-etoposide.

**Results:**

The patients’ median age was 63 years and 120 (65%) had extended stage, 75 (41%) had increased LDH serum levels and 131 (71%) an ECOG performance status was 0-1. Patients with limited stage, whose tumours expressed high *ERCC1* (*p*=0.028), *PKM2* (*p*=0.046), *TOPOI* (*p*=0.008), *TOPOIIA* (*p*=0.002) and *TOPOIIB* (p<0.001) mRNA had a shorter Progression Free Survival (PFS). In limited stage patients, high expression of *ERCC1* (*p*=0.014), *PKM2* (*p*=0.026), *TOPOIIA* (*p*=0.021) and *TOPOIIB* (*p*=0.019) was correlated with decreased median overall survival (mOS) while in patients with extended stage, only high *TOPOIIB* expression had a negative impact on Os (*p*=0.035). The favorable expression signature expression signature (low expression of *ERCC1*, PKM2, *TOPOIIA* and *TOPOIIB*) was correlated with significantly better PFS and Os in both LS-SCLC (*p*<0.001 and *p*=0.007, respectively) and ES-SCLC (*p*=0.007 and (*p*=0.011, respectively) group. The unfavorable expression signature was an independent predictor for poor PFS (HR: 3.18; *p*=0.002 and HR: 3.14; *p*=0.021) and Os (HR: 4.35; *p=*0.001and HR: 3.32; *p*=0.019) in both limited and extended stage, respectively.

**Conclusions:**

Single gene’s expression analysis as well as the integrated analysis of *ERCC1*, *PKM2, TOPOIIA* and *TOPOIIB* may predict treatment outcome in patients with SCLC. These findings should be further validated in a prospective study.

## Introduction

Small cell lung cancer (SCLC) is a biologically complex malignancy with treatment that has remained largely unchanged over the last 30 years [1]. Although the proportion of patients with SCLC has been decreased, it still accounts for approximately 15% of all lung cancer cases [2]. SCLC is characterized by early dissemination, and 70%–80% of patients have metastatic disease at diagnosis (extensive stage, ES-SCLC) while the rest present with disease limited to the thorax (limited-stage, LS-SCLC) [3]. The standard of care for SCLC is platinum-based combination chemotherapy, using either etoposide or irinotecan, in association with radiotherapy in limited stage disease (LS-SCLC). There is a typical disease trajectory with an initial good clinical response followed by rapid relapse of chemoresistant tumour and death [4]. Because surgical resection is not part of standard care, SCLC research using primary tumour tissue is limited to small diagnostic samples and, thus, the therapeutic benefits from the molecular analysis of this tumours are limited during the last 30 years [5].

Theoretically, the molecular characteristics obtained from the primary tumours have the potential to affect therapeutic decisions and to allow clinicians to select chemotherapy drugs that will give patients maximal benefit while simultaneously minimize toxicity [6]. Primary and/or acquired resistance to chemotherapy or radiotherapy is the main cause of poor outcome in patients with lung cancer [7,8]. Over the last years a growing body of evidence, from gene expression, mutational and proteomic profiling studies, as well as from *in vitro* models led to the identification of molecular markers related with resistance to platinum analogs and topoisomerase inhibitors [9].

The Excision Repair Cross Complementation group 1 (*ERCC1*) is an enzyme which plays a key role in the GG-Nucleotide Excision Repair (GG-NER) pathway which repairs DNA adducts and other DNA helix-distorting lesions including those associated with cisplatin administration [10]. The majority of studies on ERCC1 and cisplatin, in NonSmall Cell Lung Cancer (NSCLC) as well as in other solid tumours, share in common the finding that low-*ERCC1* expression is associated with a better response to platinum-based chemotherapy [11-13]. On the other hand, the results of *in vitro* studies have suggested the superiority of TC-NER pathway, in which breast cancer susceptibility gene 1 (*BRCA1*) protein is involved, to GG-NER pathway in predicting platinum resistance [14]. Modulation of *BRCA1* expression leads to modification of TC-NER and, hence, to radio- and chemo-resistance [15,16]. *BRCA1* is also involved in homologous recombination repair (HRR) and non-homologous end joining (NHEJ), in response to DNA damage and may be a regulator of mitotic spindle assembly, as *BRCA1* and b-tubulin co-localize to the microtubules of the mitotic spindle and to the centrosomes [17,18]. In addition, recent data have shown that import and export transporters involved in maintenance of copper homeostasis are also involved in the transport of cisplatin [19]. Especially, overexpression of the P-type transporter *ATP7B* conferred cisplatin resistance associated with decreased intracellular accumulation of cisplatin and carboplatin [20]. Also, the role of pyruvate kinase isoform M2 (*PKM2*) in resistance to platinum analogs is recently under extensive investigation [21,22]; *PKM2* replaces the specific isoforms (type L, R, and M1) during tumourigenesis and substitution is under the control of the transcription factor *C-MYC* [23,24]. The overexpression of MYC proteins in SCLC is largely a result of gene amplification and leads to more rapid proliferation and loss of terminal differentiation [25]. *C-MYC* overexpression occurs in 16–32% of SCLC and in 40% of cell lines established from patients whose disease progressed after chemotherapy [26,27]. Finally, several mechanisms of resistance have been suggested to involve DNA topoisomerase I and II (*TOPOI* and *TOPOII*), which are the target of several compounds, including topoisomerase-I inhibitors (such as irinotecan and topotecan) as well as topoisomerase-II inhibitors (such as etoposide and doxorubicin) [7,28,29].

This study retrospectively analyzes the predictive value on both response to treatment and survival of *BRCA1, ERCC1, ATP7B, PKM2, TOPOI, TOPΟ-IIA, TOPOIIB* and *C-MYC* mRNA levels, as they are detected by real-time PCR in SCLC patients treated with platinum-based combination chemotherapy with or without chest radiotherapy. 

## Materials and Methods

### Patients

From the database of histologically confirmed SCLC patients, treated with cisplatin and etoposide, in the Department of Medical Oncology in the University Hospital of Heraklion, we retrospectively collected all available tissue samples and the corresponding clinical data. In total, 184 formalin-fixed paraffin-embedded (FFPE) bronchoscopic or fine needle aspiration (FNA) biopsies from the primary tumours were collected. The study was approved by the institutional review board and all patients had signed informed consent for molecular analysis at the time of diagnosis. Main inclusion criteria were: histologically confirmed SCLC; first-line treatment with cisplatin or carboplatin and etoposide with or without radiotherapy and available tumour tissue. The study has been approved from the ethics and scientific committee of University Hospital of Heraklion (approval number 4456/14-5-2010) and all patients sign informed consent for their participation in the study.

### Pathological evaluation, micro-dissection and RNA extraction

All paraffin-embedded tumours were reviewed by two independent pathologists to ensure the validity of the specimen and to select the proper area for microdissection. Sections of 5µm thickness were prepared and after staining with nuclear Fast Red (Sigma-Aldrich, St Louis, MO USA) cancer cells were procured using an Eppendorf piezoelectric microdissector (Eppendorf, Hamburg, Germany). RNA extraction was performed using the trizol LS method (Invitrogen, Carlsbad, CA, USA) and 50ng of total RNA for each gene was needed to prepare cDNA, using SuperScript III reverse transcriptase (Invitrogen, Carlsbad, CA, USA) [30,31]. Relative cDNA quantification was performed using the ABI Prism 7900HT Sequence Detection System (AB). Comparative Ct method was used for gene expression quantification using *β-actin* and *PGK1* as internal reference genes and commercial RNA (Stratagene, La Jolla, CA, USA) as calibrators. Final expression values were determined as follows: 2^-(ΔCt sample-ΔCt calibrator)^, where ΔCt values of the sample and the calibrators are estimated by subtracting the Ct value of the target gene from the median of the housekeeping genes values. In all experiments, only triplicates with a standard deviation of the Ct value <0.25 were accepted. In addition, genomic DNA contamination was excluded by including non-reverse transcribed RNA as a control for all 184 patients’ samples.

Primers and probes for gene expression analysis were designed using Primer Express 2.0 Software (AB) on the basis of their Ref Seq in http://www.ncbi.nlm.nih.gov/sites/entrez?db=gene designed. The detailed sequence and hybridization data are provided in table S1.

### Statistical Considerations

Gene expression levels were quantified as continuous variable. The Pearson’s correlation coefficient analysis was used to determine correlation among different genes. Progression free survival (PFS) was calculated from the diagnosis of disease to demonstrated radiological progression or death from any cause. Overall survival (OS) was calculated from the diagnosis of disease to death from any cause. Median PFS and OS were estimated using the Kaplan-Meier curves and comparisons have been made using two-sided log-rank test. A univariate Cox regression analysis, with hazard ratios and 95% confindence intervals (CIs), was used to assess the association between each potential predictive factor and survival or PFS. These factors were then included in a multivariate Cox proportional hazards regression model with a stepwise procedure (both forward and backward) to evaluate the independent significance of different variables on survival and PFS. All results were considered as statistically significant with p<0.05 (two-sided test).

## Results

### Patients’ characteristics and clinico-pathological features

The main demographics and clinical characteristics of the study population are summarized in Table 1. Patients were predominately males (87%), with median age of 63 years and good performance status (PS; European Cooperative Oncology Group-ECOG 0-1:71%); about one third had LS-SCLC and 41% presented elevated serum levels of LDH at the time of initial diagnosis. The treatment protocol consisted of four to six three-week cycles of 100mg of eoposide per square meter on days 1, 2 and 3 and 80mg of cisplatin per square meter or carboplatin AUC 5-6 on day 1. All regimens required hydration and administration of antiemetic drugs. Sixty of LS-SCLC patients (60 out of 64; 94%) received curative radiotherapy which was started with cycle 2 of chemotherapy.

**Table 1:** Patients’ and tumours’ characteristics.

**Feature**	**Ν**	**%**
	184	
**Median Age (Range**)** years**	63 (33-78)	
≤ 65 years	98	54
**Gender**		
Male	160	87
Female	24	13
**Stage**		
Limited	64	35
Extended	120	65
**ECOG PS**		
0-1	131	71
2	53	29
**LDH @ Diagnosis**		
Normal	109	59
Elevated	75	41
**Curative Radiotherapy**		
Limited	60	33
Extended	0	0
**Relapse**		
Yes	171	93
No	13	7
**Second line Chemotherapy**		
Yes	145	79
No	39	11
**Survival Status**		
Death	169	92
Alive	15	8
**Median Progression Free Survival (Range**)	**Months**	
Extended	4.0 (0.3-16)	
Limited	8.0 (1.3-121)	
**Median Overall Survival (Range**)	**Months**	
Extended	7.6 (2.3-26.0)	
Limited	15.0 (4.6-156.0)	

Patients’ outcome was typical for SCLC. At the time of analysis and after a median follow-up of 9.1 months (min-max: 0.3-156 months), 171 disease relapses and 169 deaths have been recorded. The median OS was 15 months for patients with LS-SCLC and 8 months for those with ES-SCLC and the median PFS was 8 and 4 months, respectively (Table 1). The response to first line treatment is presented in Table 2.

**Table 2:** Response to treatment.

	**CR** n (%)	**PR** n (%)	**SD** n (%)	**PD** n (%)	**NE** n (%)
**LS-SCLC** (n=64)	13 (20)	30 (47)	7 (11)	11 (17)	3 (5)
	ORR: 43 (67)				
**ES-SCLC** (n=120)	1 (0.8)	42 (35)	18 (15)	54 (45)	5 (4.2)
	ORR: 43 (35.8)				

**CR: Complete Response, PR; Partial Response, SD: Stable Disease**,

**PD: Progressive Disease, ORR: Overall Response Rate**

### Genes’ mRNA expression and correlations

The median expression values for each gene are presented in Table S2. The correlation among several genes’ expression confirmed the biological model at the basis of the selection of the analyzed genes. Significant correlations were observed between PKM2 and *C-MYC* (r= 0.21; p= 0.015), *BRCA1* and *ERCC1* (r= 0.65; p<0.001), *TOPOI* and *TOPΟIIA* (r= 0.40; p<0.001), *TOPOI* and *TOPOIIB* (r= 0.26; p= 0.002), and between *TOPΟ-IIA* and *TOPOIIB* (r= 0.67; p<0.001). There was no any significant correlation between the studied genes and the main clinical features such as disease stage, gender, PS and LDH levels. 

### Genes’ expression and patients’ outcome

**Figure 1:**
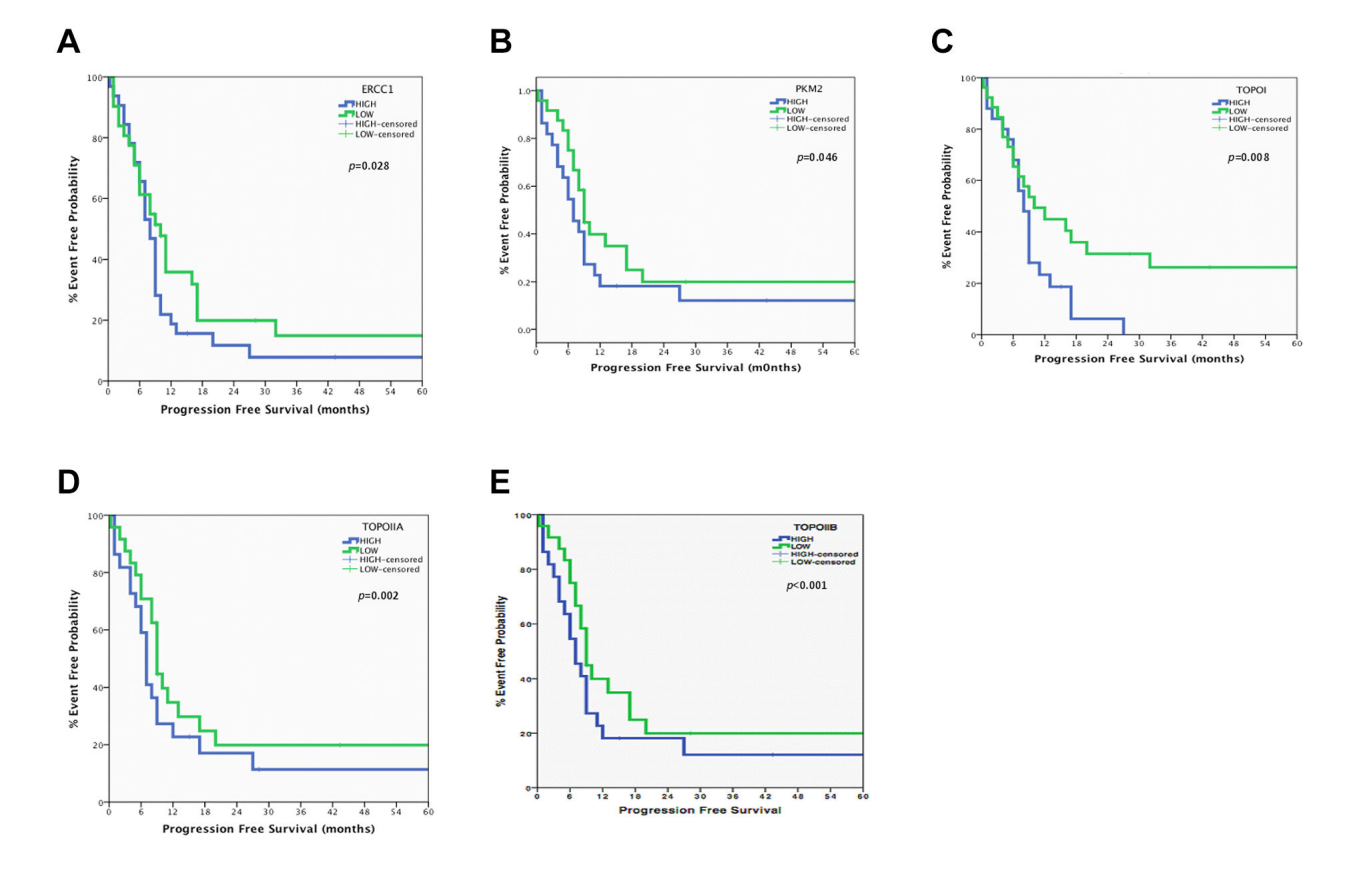
Progression Free Survival in Limited Stage-SCLC. A. *ERCC1* mRNA levels and Progression Free Survival in Limited Stage-SCLC. B. *PKM2* mRNA levels and Progression Free Survival in Limited Stage-SCLC. C. *TOPOI* mRNA levels and Progression Free Survival in Limited Stage-SCLC. D. *TOPOIIA* mRNA levels and Progression Free Survival in Limited Stage-SCLC. E. *TOPOIIB* mRNA levels and Progression Free Survival in Limited Stage-SCLC.

**Figure 2:**
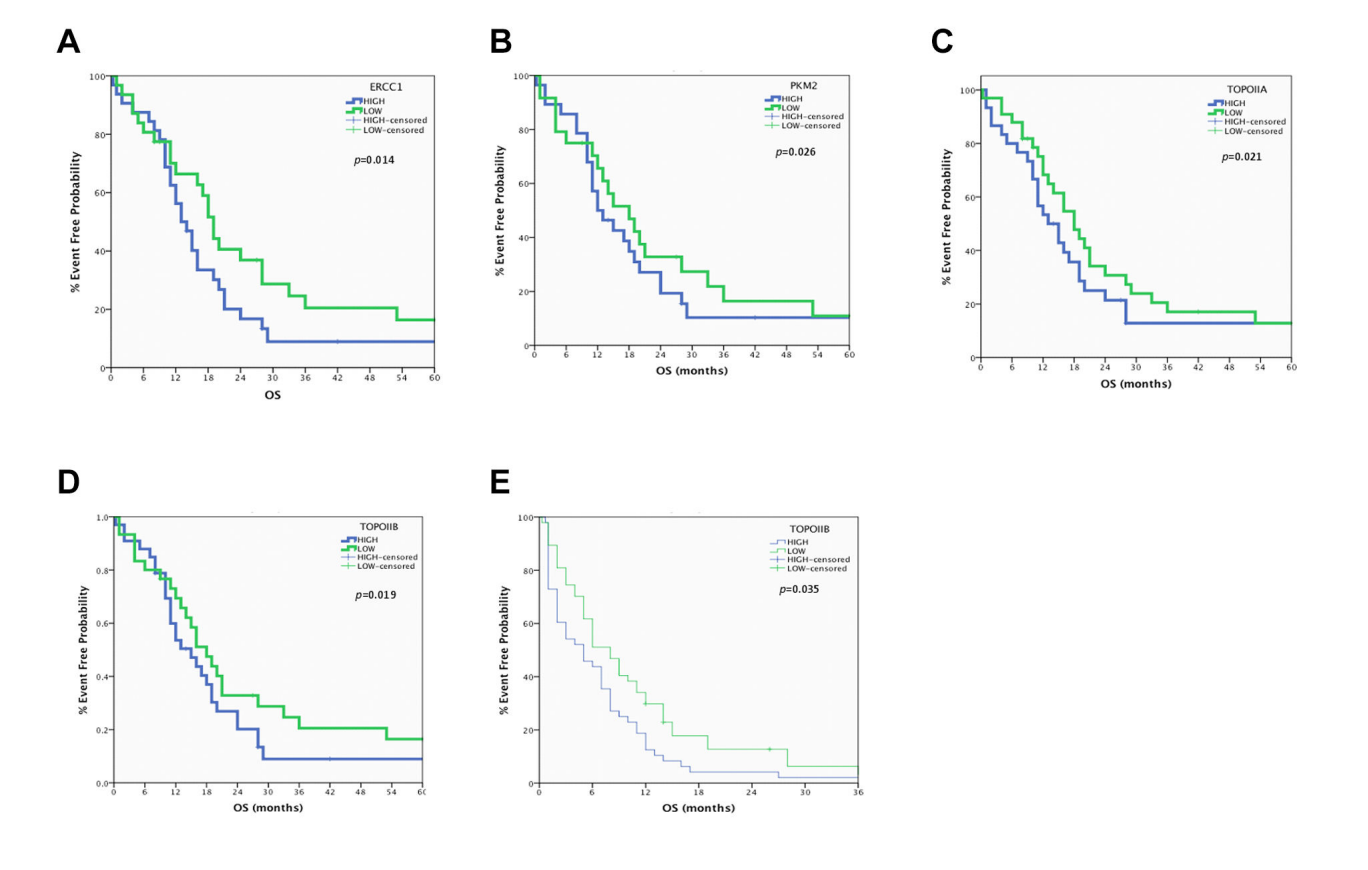
Overall Survival in Limited Stage-SCLC. A. *ERCC1* mRNA levels and Overall Survival in Limited Stage-SCLC. B. *PKM2* mRNA levels and Overall Free Survival in Limited Stage-SCLC. C. *TOPOIIA* mRNA levels and Overall Free Survival in Limited Stage-SCLC. *D*. *TOPOIIB* mRNA levels and Overall Free Survival in Limited Stage-SCLC. E. *TOPOIIB* mRNA levels and Overall Free Survival in Extended Stage-SCLC.

When the whole patients’ population was analyzed no significant correlation has been observed regarding median PFS or OS (Table S3). In contrast, when we examined separately LS-SCLC and ES-SCLC patients’ significant associations have been revealed. Indeed, in the LS-SCLC group, shorter median PFS was observed in patients with high mRNA expression of *ERCC1* (7.9 vs. 10.1 months; *p* = 0.028), *PKM2* (7.1 vs. 9.0 months; *p* = 0.046), *TOPOI* (7.8 vs. 10.2 months; *p* = 0.008), *TOPOIIA* (7 vs. 9.3 months; *p* = 0.002) and *TOPOIIB* (6.6 vs. 9.1 months; *p* <0.001) in comparison with those with low expression of these genes (Table S4 and Figure 1). Moreover, median OS was significantly decreased in patients with high mRNA expression of *ERCC1* (13.2 vs. 19.1 months; *p* = 0.014), *PKM2* (12 vs. 18.0 months; *p* = 0.026), *TOPOIIA* (13.0 vs. 18.3 months; *p* = 0.021) and *TOPOIIB* (12.8 vs. 18.4 months; *p* = 0.019) in comparison with those with low expression of these genes (Table S4 and Figure 2). No significant correlation has been observed between median PFS and OS and mRNA expression of any of the studied genes, with the exception of *TOPOIIB*, in patients with ES-SCLC (Table S5). More specifically, high mRNA expression of *TOPOIIB* was significantly associated with decreased median OS (5.0 vs. 9.2 months; *p* = 0.035) (Table S5 and Figure 2E)

**Figure 3:**
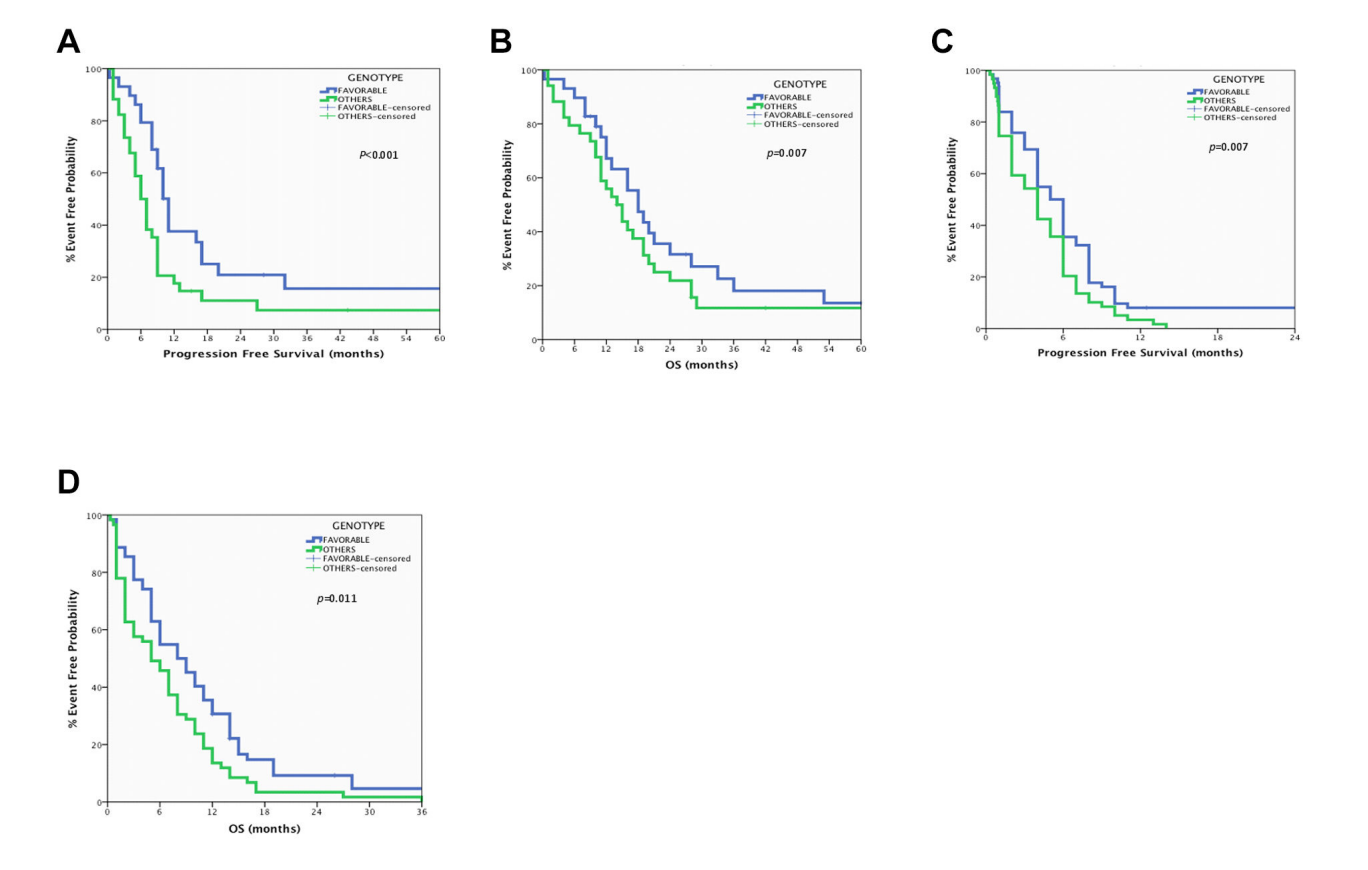
Predictive value of the expression signature (favorable: low *ERCC1, PKM2, TOPOIIA* and *TOPOIIB* mRNA levels) in SCLC. A. Correlation of expression signature with Progression Free Survival in Limited Stage-SCLC. B. Correlation of expression signature with Overall Survival in Limited Stage-SCLC. C. Correlation of expression signature with Progression Free Survival in Extended Stage-SCLC. D. Correlation of expression signature with Overall Survival in Extended Stage-SCLC.

When the expression of *ERCC1, PKM2* (sensitivity to platinum analogs) and *TOPOIIA and TOPOIIB* (sensitivity to etoposide) were combined significant correlations in both patients’ populations have been observed. The unfavorable expression signature (high mRNA expression of *ERCC1, PKM2, TOPOIIA and TOPOIIB*) in patients with LS-SCLC (n=26) was a significant predictor of shorter median PFS (6 vs. 11 months; p<0.001) and OS (13 vs. 18 months; p=0.007) when compared with those with favorable expression signature (low mRNA expression of *ERCC1, PKM2, TOPOIIA and TOPOIIB*; n=25) (Figure 3A and 3B). Similarly, in the ES-SCLC group, the patients with the unfavorable expression signature (n=53) presented significantly decreased PFS (3.8 vs. 6.1 months; p = 0.007) and OS (4.2 vs. 8.4 months; p = 0.011) in comparison with those with the favorable expression signature (n=51; Figure 3C and D)

### Multivariate analysis for PFS and OS

Univariate analysis demonstrated that in patients with LS-SCLC high *ERCC1* (HR: 1.71, 95% CI: 1.24-2.91; *p*=0.01), *PKM2* (HR: 1.64, 95% CI: 1.17-2.42; *p*=0.031), *TOPOI* (HR: 1.41, 95% CI: 1.26-1.98; *p*=0.044), TOPOIIA (HR: 1.74, 95% CI: 1.33-3.1; *p*=0.009) and *TOPOIIB* (HR: 1.82, 95% CI: 1.46-3.18; *p*=0.004) mRNA expression, the unfavourable expression signature (HR: 4.97, 95% CI: 2.74-7.61; *p*=0.001), as well as PS of 2 (HR: 1.46, 95% CI: 1.22-2.81; *p*=0.017) and elevated serum levels of LDH (HR: 1.52, 95% CI: 1.21-2.78; *p*=0.021) were significantly associated with decreased PFS (Table 3). Also, high *ERCC1* (HR: 1.63, 95% CI: 1.13-2.27; *p*=0.027), *PKM2* (HR: 1.49, 95% CI: 1.06-1.84; *p*=0.046), TOPOIIA (HR: 1.69, 95% CI: 1.2-2.34; *p*=0.018), *TOPOIIB* (HR: 1.8, 95% CI: 1.43-3.11; *p*=0.006) mRNA expression as well as the unfavourable expression signature (HR: 5.34, 95% CI: 3.85-7.62; *p*<0.0001), PS of 2 (HR: 1.58, 95% CI: 1.29-2.9; *p*=0.013) and elevated serum levels of LDH levels (HR: 1.56, 95% CI: 1.22-2.94; *p*=0.027) were associated with decreased median OS. In ES-SCLC group, only the unfavourable expression signature (HR: 3.56, 95% CI: 1.89-6.46; *p*=0.001), PS of 2 (HR: 1. 81, 95% CI: 1.17-2.83; *p*=0.023) and elevated serum levels of LDH (HR: 1.93, 95% CI: 1.43-2.94; *p*=0.019) were associated with decreased PFS, while *TOPOIIB* (HR: 1.53, 95% CI: 1.09-1.89; *p*=0.046) mRNA expression, the unfavourable expression signature (HR: 4.14, 95% CI: 1.95-6.58; *p*=0.001), PS of 2 (HR: 1.94, 95% CI: 1.62-2.58; *p*=0.017) and elevated serum levels of LDH (HR: 2.01, 95% CI: 1.61-2.64; *p*=0.013) were correlated with shorter median OS (Table 4).

**Table 3:** Univariate analysis for Progression Free Survival and Overall Survival.

	***ProgressionFreeSurvival***			***OverallSurvival***		
	**Hazard Ratio**	**95% CI**	***p*-value**			*p*-value
***Limited-StageSCLC(n=64***)						
*ERCC1* expression (High vs Low)	1.71	1.24-2.91	0.01	1.63	1.13-2.27	0.027
*PKM2* expression (High vs Low)	1. 64	1.17-2.42	0.031	1.49	1.06-1.84	0.046
*TOPOI* expression (High vs Low)	1.41	1.26-1.98	0.044	1.37	0.96-1.51	0.106
TOPOIIA expression (High vs Low)	1.74	1.33-3.1	0.009	1.69	1.2-2.34	0.018
*TOPOIIB* expression (High vs Low)	1.82	1.46-3.18	0.004	1.8	1.43-3.11	0.006
*Expression signature (unfavourable vs. favourable*)	4.97	2.74-7.61	0.001	5.34	3.85-7.62	<0.001
PS (2 vs. 0-1)	1.46	1.22-2. 81	0.017	1.58	1.29-2.9	0.013
LDH (elevated vs. normal)	1.52	1.21-2.78	0.021	1.56	1.22-2.94	0.027
***Extended-StageSCLC(n=120***)						
*TOPOIIB* expression (High vs. Low)	1.25	0.89-1.80	0.186	1.53	1.09-1.89	0.046
*Expression signature (unfavourable vs. favourable*)	3.56	1.89-6.46	0.001	4.14	1.95-6.58	0.001
PS (2 vs. 0-1)	1.81	1.17-2.83	0.023	1.94	1.62-2.58	0.017
LDH (elevated vs. normal)	1.93	1.43–2.94	0.019	2.01	1.61-2.64	0.0.13

**Table 4:** Multivariate analysis for Progression Free Survival and Overall Survival.

	***ProgressionFreeSurvival***			***OverallSurvival***		
	**Hazard Ratio**	**95% CI**	***p*-value**			*p*-value
***Limited- Stage SCLC (n=64***)						
*ERCC1* expression (High vs Low)	1.34	0.96-1.89	0.091	1.33	0.93-1.77	0.104
*PKM2* expression (High vs Low)	1. 32	0.93-1.82	0.111	1.39	0.96-1.83	0.086
*TOPOI* expression (High vs Low)	1.29	0.86-1.78	0.178	1.26	0.81-1.61	0.215
TOPOIIA expression (High vs Low)	1.62	1.13-2.41	0.039	1.49	0.97-2.12	0.081
*TOPOIIB* expression (High vs Low)	1.79	1.40-2. 88	0.022	1.77	1.41-2.91	0.016
*Expression signature (unfavourable vs. favourable*)	3.18	1.94-4.85	0.002	4.35	2.76-6.08	0.001
PS (2 vs. 0-1)	1.38	1.14-2. 63	0.02	1.45	1.22-2.64	0.016
LDH (elevated vs. normal)	1.6	1.32-2.94	0.017	1.63	1.29-3.01	0.011
***Extended-StageSCLC(n=120***)						
*TOPOIIB* expression (High vs Low)	1.11	0.74-1.63	0.29	1.44	0.98-1.83	0.064
*Expression signature (unfavourable vs. favourable*)	3.14	1.76-4.82	0.021	3.32	1.91-5.32	0.019
PS (2 vs. 0-1)	1.76	1.19-2.78	0.031	1.88	1.58-2.47	0.024
LDH (elevated vs. normal)	1.84	1.41–2.77	0.022	1.92	1.56-2.44	0.0.19

Cox proportional hazard analysis revealed that in patients with LS-SCLC high mRNA expression of TOPOIIA (HR: 1.62, 95% CI: 1.13-2.41; *p*=0.039) and *TOPOIIB* (HR: 1.79, 95% CI: 1.40-2.88; *p*=0.022) as well as unfavourable expression signature (HR: 3.18, 95% CI: 1.94-4.85; *p*=0.002), PS of 2 (HR: 1. 38, 95% CI: 1.14-2.63; *p*=0.02) and elevated serum levels of LDH (HR: 1.6, 95% CI: 1.32-2.94; *p*=0.017) emerged as independent factors associated with decreased PFS (Table 4). Similarly, *TOPOIIB* (HR: 1.77, 95% CI: 1.41-2.91; *p*=0.016) mRNA expression, unfavourable expression signature (HR: 4.35, 95% CI: 2.76-6.08; *p*=0.001) ), PS of 2 (HR: 1. 45, 95% CI: 1.22-2.64; *p*=0.016) and elevated serum levels of LDH levels (HR: 1.63, 95% CI: 1.29-3.01; *p*=0.011) were emerged as independent factors correlated with decreased median OS in the same group of patients (Table 4). In the group of patients with ES-SCLC only the unfavourable expression signature (HR: 3.14, 95% CI: 1.76-4.82; *p*=0.021), PS of 2 (HR: 1.76, 95% CI: 1.19-2.78; *p*=0.031) and elevated serum levels of LDH (HR: 1.84, 95% CI: 1.41-2.77; *p*=0.022) emerged as independent factors associated with decreased PFS (Table 4). Similarly, unfavourable expression signature (HR: 3.32, 95% CI: 1.91-5.32; *p*=0.019), PS of 2 (HR: 1. 88, 95% CI: 1.58-2.47; *p*=0.024) and elevated serum levels of LDH (HR: 1.92, 95% CI: 1.56-2.44; *p*=0.019), as well as *TOPOIIB* mRNA expression (HR: 1.44, 95% CI: 0.98-1.83; *p*=0.064) were revealed as independent factors associated with shorter median OS (Table 4).

## Discussion

Over the past decades, there has been a modest improvement in survival of patients with SCLC, but the percentage of long-term survivors remains dismal. Due to the difficulty in obtaining sufficient and adequate tumour material, only few small studies have thus far been performed in SCLC and this disease remains an important target for treatment and research. In the present study which, in the best of our knowledge is the largest one, we analyzed the expression of several related genes with treatment response and we found a potential predictive model in SCLC patients treated with cisplatin and etoposide.

For the first time in 1999, new biological predictive markers were observed to have an impact on response to chemotherapy and survival of patients with SCLC. Consistent with our findings, Dingemans et al reported that *TOPOIIB* expression was predictive for response to chemotherapy, with higher response rates in patients with low *TOPOIIB* levels while high *TOPOIIA* expression predicted for shorter survival [7]. In agreement with these findings, we observed that LS-SCLC patients with low mRNA expression levels of *TOPOIIA* and *TOPOIIB* had significantly better PFS (*p*=0.002 and *p*<0.001, respectively) and OS (*p*=0.021 and *p*=0.019, respectively), while *TOPOIIB* was correlated with decreased median OS in ES-SCLC (*p*=0.035). The most of the *in vitro* studies have reported a close correlation between the expression of *TOPOIIA* and sensitivity to drugs. Also, the higher expression of this enzyme in SCLC has been reported as a possible reason for the higher chemosensitivity [32]. In contrast our results are in the opposite direction and this discrepancy may be attributed to methodological issues (protein versus mRNA expression, cell lines versus human cancer samples etc); in addition, it might be partially explained by the fact that, in our study, *TOPOII* inhibitors were administered together with cisplatin. The findings regarding the expression of *TOPOIIB* are also supported by other studies in SCLC [33].

In addition, *TOPOI* overexpression has been correlated with decreased PFS (HR: 1.41; *p*=0.044). Indeed, augmented expression of *TOPOI* has been described in various types of tumours [34]. For instance, ovarian cancer cases treated with platinum-based drugs but not with topotecan with increased expression of *TOPOIA* demonstrated a significant shorter overall survival, indicating that on cases with augmented expression of *TOPOIA* application of topotecan should be considered as it might change the outcome in this group of patients [35], but the biological rationale is lacking.

The current study also demonstrated that LS-SCLC patients with high expression of *ERCC1* and *PKM2* had shorter median PFS (*p*=0.028 and *p*=0.046, respectively) and OS (*p*=0.014 and *p*=0.026, respectively), while patients with low levels of *TOPOI* presented longer PFS (*p*=0.008) but not OS (*p*=0.41) compared with those with high mRNA expression. These findings are in agreement with those from recently reported studies which demonstrated that the protein or mRNA expression of *ERCC1* and *TOPOI* in SCLC is predictive of treatment efficacy [33,36]. Sereno et al have demonstrated that *TOPOI* mRNA analysis can predict cispaltin response and prognosis in SCLC patients [36]. Simultaneously, since expression of *TOPOIA* has been documented as a significant predictive index in camptothecin based therapy, tumours with high expression of *TOPOI* may be more amenable to this type of treatment [35]. Also, Ceppi et al, quantified ERCC1, RRM1, and *TOPOII* mRNA expression in 85 SCLC patients treated with platinum/etoposide [33]; they found that *TOPOII* expression was associated with better response in LS-SCLC patients, while patients of the same stage of disease and low *ERCC1* mRNA levels had significantly longer survival. Τhe multivariate analysis demonstrated that *ERCC1* expression was an independent prognostic factor for survival in LS-SCLC [33]. Similarly, Lee et al, reported that the protein expression of *ERCC1* in 77 SCLC patients treated with platinum-based doublets, was associated with poor OS, especially in patients with LS-SCLC indicating that high expression of *ERCC1* can be a prognostic biomarker for this group of patients [37]. Finally, Chiappori et al, using an immunofluorescence-based automated quantitative technique, scoring *RRM1, ERCC1* and *TOPOII* levels in tumour specimens, reported that *TOPOII* mRNA expression predicted for better response while *ERCC1* mRNA expression was the only independent prognostic factor for survival; conversely, there was no prognostic or predictive role for any of these genes in ES-SCLC [38]. These findings strongly suggest that LS-SCLC seems to represent a disease which may be biologically distinct from ES-SCLC based on the affected molecular mechanism.

Another important finding in the present study is that the integrated analysis of *ERCC1*, *PKM2* (related with resistance to cisplatin), *TOPOIIA* and *TOPOIIB* (related with resistance to etoposide) was able to confirm that the group of patients with the favorable expression signature (low expression of all genes) presented an improved OS and PFS when compared with those with the unfavorable expression signature. In fact, in LS-SCLC patients with the favorable expression signature, a PFS of almost one year (*p*<0.001) and an OS of 18 months (*p*=0.007) was observed, whereas ES-SCLC patients with low expression of the four genes achieved a PFS of 6.1 months (*p*=0.007) and an OS of 8.4 months (*p*=0.011) which were significantly higher compared to patients with the unfavorable expression signature (3.8 months and 4.2 for PFS and OS, respectively).

Despite that, the promising results of the present study should be interpreted with caution due to the limitation of this type of research and not definitive conclusions could be made. The study was retrospective, lacks a validation group and although has included the larger number of patients’ samples reported ever, the total number of enrolled patients remains relatively small. However, we consider that this type of research may identify subgroups of patients with substantial benefit from the standard chemotherapy and radiotherapy for SCLC and may lead to the design of a prospective clinical trial in SCLC, where the predictive powers of these biomarker expression levels will be tested and validated prospectively. Finally, this study indicates that even in malignancies with limitations in tissue availability, such as SCLC, combinatory efforts may provide adequate samples for molecular analysis and contribute by that to the goal of “individualized” treatment. 

## Supporting Information

Table S1 **Sequence of the primers and probes of all reference and target genes.**
(DOC)

Table S2 **Genes’ expression values.**
(DOC)

Table S3 **Whole patients’ population: Correlation of genes’ expression value with Progression Free Survival and Overall Survival.**
(DOCX)

Table S4 **LS-SCLC#: Correlation of genes’ expression value and Progression Free Survival and Overall Survival.**
(DOCX)

Table S5 **ES-SCLC#: Correlation of genes’ expression value and Progression Free Survival and Overall Survival.**
(DOCX)
