# Supplementary material for: Predictive Value of BRCA1, ERCC1, ATP7B, PKM2, TOPOI, TOPΟ-IIA, TOPOIIB and C-MYC Genes in Patients with Small Cell Lung Cancer (SCLC) Who Received First Line Therapy with Cisplatin and Etoposide
Source: PLoS One. 2013 Sep 13;8(9):e74611. doi: 10.1371/journal.pone.0074611 (PMC3772910; doi:10.1371/journal.pone.0074611)
Supplement: Table S1 — Sequence of the primers and probes of all reference and target genes. (DOC) [file pone.0074611.s001.doc]

**Supplementary Table S1.** Sequence of the primers and probes of all reference and target genes

| **Gene** | **Forward Primer** | **5’-labeled (FAM) probe** | **Reverse Primer** |
| --- | --- | --- | --- |
| ***β-actin*** | 5’-GGC ACC CAG CAC AAT GAA G-3’ | **5’** TCA AGA TCA TTG CTC CTC CTG AGC GC**--3** | **’**5’-GCC GAT CCA CAC GGA GTA CT-3’ |
| ***PGK1*** | 5’- GGCTGGATGGGCTTGGA –3’ | 5-TGTGGTCCTGAAAGCAGCAAGAAGTATGC -3’ | 5’-TCTGCTTAGCCCGAGTGACA-3 |
| ***BRCA1*** | 5’-GGC TAT CCT CTC AGA GTG ACA TTT TA-3’ | 5’-CCA CTC AGC AGA GGG-3’ | 5’-GCT TTA TCA GGT TAT GTT GCA TGG T-3’ |
| ***ERCC1*** | 5’-GGG AAT TTG GCG ACG TAA TTC-3’ | 5’-CAC AGG TGC TCT GGC CCA GCA CAT A-3’ | 5’-GCG GAG GCT GAG GAA CAG-3’ |
| ***PKM2*** | 5’-GCC ATA ATC GTC CTC ACC AAG T-3’ | 5’-CAG GTC TGC TCA CCA GG -3’ | 5’-GCA CGT GGG CGG TAT CTG-3’ |
| ***MYC*** | **5’-GAG CCC CTG GTG CTC CAT-3’** | 5’-AGG AGA CAC CGC CCA C-3’ | 5’-TCA TCT TCT TGT TCC TCC TCA GAG T-3’ |
| ***ATP7b*** | **5’-CAT CCT GTG TGT CTA ACA TAG AAA GGA-3’** | **5’- CTG CAG AAA GAA GCT GGT-3’** | **5’-AGG CAA CCA ACA CGG AGA GA-3’** |
| ***TOPO-I*** | 5’-GAG AGC TGT AGC CCT GTA CTT CAT C-3’ | 5’-CAA GCT TGC TCT GAG AGC AGG CAA TG-3’ | 5’- CAG TGT CCG CTG TTT CTC CTT -3’ |
| ***TOPO-IIa*** | 5’-CAG AGC TTC CCG TCA GAA CAT-3’ | 5’-CAG ACA TAC AAA GAA CAA GT-3’ | 5’-GTG CCA TTC AAC ATG GGT TCT A-3’ |
| ***TOPO-IIb*** | 5’- TAT ATT GGG TCA GTG GAG CCA TT-3’ | 5’-CGC AGT TCA TGT GGG TG-3’ | 5’- TGC AAT TCA TTC CTA CAT CTT CAT C-3’ |
