# Supplementary material for: Predictive Value of BRCA1, ERCC1, ATP7B, PKM2, TOPOI, TOPΟ-IIA, TOPOIIB and C-MYC Genes in Patients with Small Cell Lung Cancer (SCLC) Who Received First Line Therapy with Cisplatin and Etoposide
Source: PLoS One. 2013 Sep 13;8(9):e74611. doi: 10.1371/journal.pone.0074611 (PMC3772910; doi:10.1371/journal.pone.0074611)
Supplement: Table S2 — Genes’ expression values. (DOC) [file pone.0074611.s002.doc]

**Supplementary Table S2:** Genes’ expression values

|  |  | **All patients** | **LS-SCLC^#^** | **ES-SCLS@** | ***p* value** |
| --- | --- | --- | --- | --- | --- |
|  | ***No of patients (%)*** | **184 (100)** | **64 (35)** | **120 (52)** |  |
| ***BRCA1*** | *Expression value*  *Median (range)* | 6.99  (0.26-44.04) | 6.89  (0.26-42.15) | 7.01  (0.52-44.04) | *0.81* |
|  | *High expression* | 92 (50) | 32(50) | 60 (50) |  |
|  | *Low expression* | 92 (50) | 32(50) | 60 (50) |  |
| ***ERCC1*** | *Expression value*  *Median (range)* | 12.27  (0.02-273.77) | 11.49  (0.02-216.7) | 12.51  (0.29-273.77) | *0.76* |
|  | *High expression* | 92 (50) | 32(50) | 60 (50) |  |
|  | *Low expression* | 92 (50) | 32(50) | 60 (50) |  |
| ***PKM2*** | *Expression value*  *Median (range)* | 20.46  (6.5-117.22) | 20.23  (6.5-115.42) | 20.54  (6.82-111.22) | *1.0* |
|  | *High expression* | 92 (50) | 32(50) | 60 (50) |  |
|  | *Low expression* | 92 (50) | 32(50) | 60 (50) |  |
| ***MYC*** | *Expression value*  *Median (range)* | 0.33  (0.01-21.33) | 0.35  (0.01-21.33) | 0.33  (0.02-20.87) | *1.0* |
|  | *High expression* | 92 (50) | 32(50) | 60 (50) |  |
|  | *Low expression* | 92 (50) | 32(50) | 60 (50) |  |
| ***ATP7b*** | *Expression value*  *Median (range)* | 0.4  (0.01-1.87) | 0. 37  (0.01-1.54) | 0. 42  (0.03-1.87) | *0.69* |
|  | *High expression* | 92 (50) | 32(50) | 60 (50) |  |
|  | *Low expression* | 92 (50) | 32(50) | 60 (50) |  |
| ***TOPO-I*** | *Expression value*  *Median (range)* | 2.12  (1.02-26.12) | 2.23  (1.02-26.12) | 2.04  (1.09-21.98) | *0.83* |
|  | *High expression* | 92 (50) | 32(50) | 60 (50) |  |
|  | *Low expression* | 92 (50) | 32(50) | 60 (50) |  |
| ***TOPO-IIa*** | *Expression value*  *Median (range)* | 68.38  (4.43-226.3) | 66.16  (4.43-211.5) | 70.41  (4.82-226.3) | *0.57* |
|  | *High expression* | 92 (50) | 32(50) | 60 (50) |  |
|  | *Low expression* | 92 (50) | 32(50) | 60 (50) |  |
| ***TOPO-IIb*** | *Expression value*  *Median (range)* | 10.96  (2.14-103.28) | 10.63  (2.02-101.54) | 11.24  (2.14-103.28) | *0.81* |
|  | *High expression* | 92 (50) | 32(50) | 60 (50) |  |
|  | *Low expression* | 92 (50) | 32(50) | 60 (50) |  |

^#^Limited Stage Small Cell Lung Cancer

@Extended Stage Small Cell Lung Cancer
