# Supplementary material for: Predictive Value of BRCA1, ERCC1, ATP7B, PKM2, TOPOI, TOPΟ-IIA, TOPOIIB and C-MYC Genes in Patients with Small Cell Lung Cancer (SCLC) Who Received First Line Therapy with Cisplatin and Etoposide
Source: PLoS One. 2013 Sep 13;8(9):e74611. doi: 10.1371/journal.pone.0074611 (PMC3772910; doi:10.1371/journal.pone.0074611)
Supplement: Table S3 — Whole patients’ population: Correlation of genes’ expression value with Progression Free Survival and Overall Survival. (DOCX) [file pone.0074611.s003.docx]

**Supplementary Table S3.** Whole patients’ population: Correlation of genes’ expression value with Progression Free Survival and Overall Survival

|  | **Progression Free Survival (months)** | | | **Overall Survival (months)** | | |
| --- | --- | --- | --- | --- | --- | --- |
| **Gene** | **Median** | **95% CI*** | ***p* value** | **Median** | **95% CI*** | ***p* value** |
| ***BRCA1*** |  |  |  |  |  |  |
| Low | 6.1 | 5.28-6.71 | 0.71 | 9.2 | 6.4-11.2 | 0.78 |
| High | 5.2 | 3.87-6.13 |  | 8.6 | 5.9-10.8 |  |
| ***ERCC1*** |  |  |  |  |  |  |
| Low | 6.3 | 5.13-7.42 | 0.49 | 9.4 | 6.6-11.8 | 0.27 |
| High | 5.8 | 4.77-6.86 |  | 8.2 | 5.4-10.3 |  |
| ***PKM2*** |  |  |  |  |  |  |
| Low | 6.0 | 4.59-7.41 | 0.44 | 9.3 | 6.5-11.7 | 0.36 |
| High | 4.0 | 2.68-5.31 |  | 8.6 | 5.8-11.0 |  |
| ***MYC*** |  |  |  |  |  |  |
| Low | 6.0 | 4.23-7.77 | 0.18 | 9.1 | 6.5-11.0 | 0.41 |
| High | 4.0 | 2.58-5.42 |  | 8.8 | 6.1-10.9 |  |
| ***ATP7b*** |  |  |  |  |  |  |
| Low | 6.0 | 4.9-7.1 | 0.79 | 9.0 | 6.3-10.7 | 0.90 |
| High | 6.0 | 4.29-7.7 |  | 9.0 | 6.2-11.2 |  |
| ***TOPOI*** |  |  |  |  |  |  |
| Low | 6.0 | 4.29-7.7 | 0.34 | 9.2 | 6.4-10.8 | 0.62 |
| High | 5.1 | 3.77-6.23 |  | 8.7 | 6.2-10.7 |  |
| ***TOPOIIA*** |  |  |  |  |  |  |
| Low | 6.1 | 4.64-7.36 | 0.13 | 9.2 | 6.4-11.0 | 0.60 |
| High | 4.0 | 2.77-5.23 |  | 8.7 | 6.3-10.5 |  |
| ***TOPOIIB*** |  |  |  |  |  |  |
| Low | 6.2 | 4.73-7.27 | 0.41 | 9.6 | 6.9-11.4 | 0.23 |
| High | 3.8 | 2.77-5.22 |  | 8.5 | 6.1-10.6 |  |

***CI: confidence interval**
