# Supplementary material for: Predictive Value of BRCA1, ERCC1, ATP7B, PKM2, TOPOI, TOPΟ-IIA, TOPOIIB and C-MYC Genes in Patients with Small Cell Lung Cancer (SCLC) Who Received First Line Therapy with Cisplatin and Etoposide
Source: PLoS One. 2013 Sep 13;8(9):e74611. doi: 10.1371/journal.pone.0074611 (PMC3772910; doi:10.1371/journal.pone.0074611)
Supplement: Table S4 — LS-SCLC#: Correlation of genes’ expression value and Progression Free Survival and Overall Survival. (DOCX) [file pone.0074611.s004.docx]

**Supplementary Table S4.** LS-SCLC^#^: Correlation of genes’ expression value and Progression Free Survival and Overall Survival

|  | **Progression Free Survival (months)** | | | **Overall Survival (months)** | | |
| --- | --- | --- | --- | --- | --- | --- |
| **Gene** | **Median** | **95% CI*** | ***p* value** | **Median** | **95% CI*** | ***p* value** |
| ***BRCA1*** |  |  |  |  |  |  |
| Low | 9.0 | 7.4-11.9 | 0.84 | 15.6 | 10.9-21.1 | 0.38 |
| High | 8.2 | 6.2-9.8 |  | 13.8 | 10.1-17.8 |  |
| ***ERCC1*** |  |  |  |  |  |  |
| Low | 10.1 | 7.5-10.4 | 0.028 | 19.1 | 16.5-21.6 | 0.014 |
| High | 7.9 | 6.6-9.3 |  | 13.2 | 9.7-16.3 |  |
| ***PKM2*** |  |  |  |  |  |  |
| Low | 9.0 | 7.4-10.5 | 0.046 | 18.0 | 15.6-25.4 | 0.026 |
| High | 7.1 | 4.6-8.9 |  | 12.0 | 6.9-14.1 |  |
| ***MYC*** |  |  |  |  |  |  |
| Low | 8.9 | 7.2-10.7 | 0.092 | 17.8 | 13.7-26.1 | 0.095 |
| High | 7.3 | 4.8-9.1 |  | 12.2 | 6.2-16.7 |  |
| ***ATP7B*** |  |  |  |  |  |  |
| Low | 9.0 | 7.6-11.1 | 0.93 | 15.3 | 10.6-21.0 | 0.91 |
| High | 8.8 | 7.3-10.7 |  | 14.8 | 10.3-20.7 |  |
| ***TOPOI*** |  |  |  |  |  |  |
| Low | 7.8 | 6.6-8.3 | 0.008 | 13.9 | 9.0-19.1 | 0.41 |
| High | 10.2 | 7.9-16.2 |  | 16.1 | 9.8-22.2 |  |
| ***TOPOIIA*** |  |  |  |  |  |  |
| Low | 7.0 | 4.6-7.4 | 0.002 | 18.3 | 12.8-23.2 | 0.021 |
| High | 9.3 | 7.7-10.9 |  | 13.0 | 7.8-17.1 |  |
| ***TOPOIIB*** |  |  |  |  |  |  |
| Low | 6.6 | 4.2-8.7 | <0.001 | 18.4 | 16.4-21.8 | 0.019 |
| High | 9.1 | 7.9-11.5 |  | 12.8 | 9.6-16.7 |  |

***CI: confidence interval, ^#^ Limited Stage Small Cell Lung Cancer**
