# Supplementary material for: Predictive Value of BRCA1, ERCC1, ATP7B, PKM2, TOPOI, TOPΟ-IIA, TOPOIIB and C-MYC Genes in Patients with Small Cell Lung Cancer (SCLC) Who Received First Line Therapy with Cisplatin and Etoposide
Source: PLoS One. 2013 Sep 13;8(9):e74611. doi: 10.1371/journal.pone.0074611 (PMC3772910; doi:10.1371/journal.pone.0074611)
Supplement: Table S5 — ES-SCLC#: Correlation of genes’ expression value and Progression Free Survival and Overall Survival. (DOCX) [file pone.0074611.s005.docx]

**Supplementary Table S5.** ES-SCLC^#^: Correlation of genes’ expression value and Progression Free Survival and Overall Survival

|  | **Progression Free Survival (months)** | | | **Overall Survival (months)** | | |
| --- | --- | --- | --- | --- | --- | --- |
| **Gene** | **Median** | **95% CI*** | ***p* value** | **Median** | **95% CI*** | ***p* value** |
| ***BRCA1*** |  |  |  |  |  |  |
| Low | 4.2 | 2.7-5.6 | 0.85 | 7.6 | 6.8-9.9 | 0.53 |
| High | 3.9 | 2.2-5.0 |  | 6.9 | 4.7-8.5 |  |
| ***ERCC1*** |  |  |  |  |  |  |
| Low | 5.1 | 4.1-6.0 | 0.12 | 8.0 | 6.7-10.6 | 0.14 |
| High | 3.9 | 3.2-4.7 |  | 6.8 | 3.7-8.2 |  |
| ***PKM2*** |  |  |  |  |  |  |
| Low | 4.0 | 2.7-5.5 | 0.51 | 7.9 | 6.7-10.7 | 0.34 |
| High | 4.0 | 2.4-5.3 |  | 7.2 | 4.0-8.3 |  |
| ***MYC*** |  |  |  |  |  |  |
| Low | 4.1 | 2.6-5.7 | 0.82 | 7.8 | 6.7-10.5 | 0.46 |
| High | 3.9 | 2.8-5.1 |  | 7.4 | 4.1-8.1 |  |
| ***ATP7B*** |  |  |  |  |  |  |
| Low | 4.0 | 2.6-5.1 | 0.94 | 7.6 | 6.3-9.8 | 0.96 |
| High | 4.0 | 2.3-4.7 |  | 7.6 | 6.0-9.7 |  |
| ***TOPOI*** |  |  |  |  |  |  |
| Low | 4.2 | 2.8-6.3 | 0.64 | 7.8 | 7.0-10.4 | 0.70 |
| High | 3.9 | 2.4-5.2 |  | 7.4 | 6.8-10.1 |  |
| ***TOPOIIA*** |  |  |  |  |  |  |
| Low | 4.1 | 2.9-5.9 | 0.23 | 8.0 | 6.5-10.9 | 0.31 |
| High | 3.9 | 2.1-5.0 |  | 6.9 | 3.9-8.5 |  |
| ***TOPOIIB*** |  |  |  |  |  |  |
| Low | 5.1 | 3.9-6.1 | 0.11 | 9.2 | 7.3-11.8 | 0.035 |
| High | 3.9 | 2.3-5.7 |  | 5.0 | 2.6-6.7 |  |

***CI: confidence interval, ^#^ Extended Stage Small Cell Lung Cancer**
